# Anterior to posterior preoperative risk assessment of abdominal thickness compared to BMI in ventral hernias

**DOI:** 10.1007/s10029-026-03620-y

**Published:** 2026-03-24

**Authors:** Paul J. Brosnihan, Erik T. Pihl, Austin E. Reifel, Patrick K. Choi, Kathryn T. Chen, Ashkan Moazzez, Junko J. Ozao-Choy

**Affiliations:** 1 Department of Surgery, Harbor UCLA Medical Center, Box 461 , 1000 W Carson Street, Torrance, CA USA; 2 Department of Radiology, Harbor UCLA Medical Center, Torrance, CA USA

**Keywords:** Ventral hernia, BMI, Hernia recurrence, Visceral obesity, Elective hernia repair

## Abstract

**Background:**

Obesity is a known risk factor for recurrence following ventral hernia repair. BMI is often used to define obesity, and we have previously shown a BMI > 33.67 associated with higher recurrence. In 2023, AMA policy highlighted BMI as an imperfect measurement of obesity and recommended limitations to its use. This study’s objective was to evaluate the association between anterior-to-posterior abdominal wall depth (APD) in CT measurements with hernia recurrence as an alternative to BMI.

**Methods:**

Data was retrospectively collected for patients from a county healthcare system, undergoing elective ventral hernia repair from 2014 to 2020 with fascial defects > 4 cm and preoperative CT scans. CART analysis was performed to determine the APD threshold for recurrence. Receiver operating characteristic (ROC) curve analysis was performed to compare APD and BMI as predictors of recurrence. Kaplan–Meier analysis was used to depict the recurrence-free survival period.

**Results:**

267 patients met our inclusion criteria. Mean APD at L4 was 27.67 cm. APD of 29.7 cm was determined as the threshold for recurrence. Area under the curve for APD > 29.7 cm and BMI > 33.67 were 0.617 (*p* = 0.046) and 0.577 (*p*=0.189) respectively. Five-year recurrence free survival was 70% for APD ≤ 29.7 cm and 37% for APD > 29.7 cm.

**Conclusion:**

In our study, the use of APD CT measurements provided an objective, reproducible, and rapid method to augment preoperative evaluation for visceral obesity and the risk for hernia recurrence that was not reliant on traditional BMI, and, in fact, improved upon a simple BMI threshold.

## Introduction

Representing over $9.7 billion dollars in estimated annual healthcare costs in the United States, ventral hernia repair is one of the most common surgeries performed by general surgeons [1]. Despite its widespread prevalence, reported ventral hernia recurrence rates range up to 54%, contributing to morbidity, mortality, increased healthcare expenditure, poor patient satisfaction, and additional surgical interventions [2]. – [3] A recurrence reduction of 1% is estimated to save the healthcare system over $130 million per year [1, 4]. Given the high recurrence rates, optimizing patient selection and addressing modifiable risk factors are critical to improving surgical outcomes. Among these risk factors, obesity plays a particularly significant role. Obesity, with increased intra-abdominal pressure and impaired wound healing, is a well-established risk factor for ventral hernia recurrence [3, 5]. Additionally, it has been shown to be an independent risk factor for increased morbidity, surgical site infections, and both incarcerated and strangulated hernias [3, 6, 7] With the prevalence of obesity in U.S. adults exceeding 42%, it is one of the key factors surgeons must mitigate in their preoperative evaluation of any patient with a ventral hernia [8]. 

Body Mass Index (BMI) is the most commonly used measure and surrogate of obesity in the U.S., and various BMI thresholds have been proposed for optimizing patient selection in elective ventral hernia repair. However, BMI does not account for body composition or fat distribution, such as visceral versus lower extremity adiposity, and in 2023 the American Medical Association (AMA) acknowledged BMI’s limitations and recommended more comprehensive assessment methods [9]. – [10] Previous literature by our group identified a BMI threshold of > 33.67 as a predictor of increased hernia recurrence, yet emerging evidence suggests that visceral adiposity is more strongly correlated with surgical complications and hernia recurrence than overall BMI [11–14]. Our group sought alternative strategies for assessing the impact of visceral adiposity. We utilized readily available computerized tomographic (CT) imaging to assess the skin-to-skin anterior-to-posterior depth (APD) of the person in centimeters at the umbilicus, measured at the 4th lumbar vertebra. The APD has been previously validated within radiological literature for measuring visceral adiposity [15–22]. APD may serve as a more precise metric for assessing visceral adiposity and identifying patients at higher risk for hernia recurrence. The primary objective of this study is to evaluate the association between APD and hernia recurrence and to determine the threshold at which recurrence risk increases, based on preoperative CT scan measurements. Our secondary objective is to compare the predictive accuracy of APD versus BMI for hernia recurrence, with the hypothesis that APD will serve as a stronger and more reliable predictor of recurrence following ventral hernia repair.

## Methods

This study was deemed exempt from requiring institutional review board approval by the Lundquist Institute at Harbor-UCLA. Patient data were retrospectively gathered from electronic medical records for individuals who underwent elective ventral or incisional hernia repair between 2016 and 2020 within a county-wide healthcare network. This study encompassed cases from five hospitals in the Los Angeles county network, three of which are university-adjacent inpatient medical centers and two of which provide community-based ambulatory surgery. This research did not receive any specific grant from funding agencies in the public, commercial, or not-for-profit sectors.

### Inclusion and exclusion criteria

The study focused exclusively on adult patients (≥ 18 years old) who underwent elective ventral or incisional hernia repair. Patient identification was based on the following Current Procedural Terminology (CPT) codes for both open (49560, 49561, 49565, 49566, 49570, 49572) and laparoscopic procedures: (49652, 49653, 49654, 49655, 49656, 49656, and 49657). Diagnoses were validated through a review of electronic medical records. Patients were included if their primary procedure involved elective ventral or incisional hernia repair, with fascial defects of at least 4 cm based on width or length to exclude small hernias such as umbilical hernias which are sometimes repaired primarily. Defect size was confirmed by reviewing preoperative CT imaging that must have been done in the year preceding surgery. Exclusion criteria included patients who solely underwent umbilical or Spigelian hernia repair due to differing pathophysiology, those who had obstruction or strangulation at the time of surgery who frequently will undergo other concurrent procedures such as bowel resection and may not undergo mesh repair, as well as those with missing defect size documentation or absent preoperative CT imaging. Incarcerated hernias were included, because these repairs can still be performed under optimized elective conditions with standard mesh-based techniques.

### Clinical data

Collected patient data included demographic details (age at surgery, sex, and ethnicity categorized as Asian or Pacific Islander, Black, Hispanic, non-Hispanic White, or unspecified), comorbidities (BMI, tobacco use, diabetes, hypertension, cardiovascular disease, pulmonary disease, chronic kidney disease, and cirrhosis), and hernia characteristics (defect size, primary or recurrent status, reducibility, or incarceration). Operative variables included mesh type, mesh placement (onlay, **retrorectus**, underlay, or intraperitoneal), and surgical approach (open or laparoscopic). Recurrence was defined as any instance where hernia reappearance was confirmed via imaging or diagnosed on physical exam during a follow-up encounter.

### Imaging analysis

**Fig. 1:**
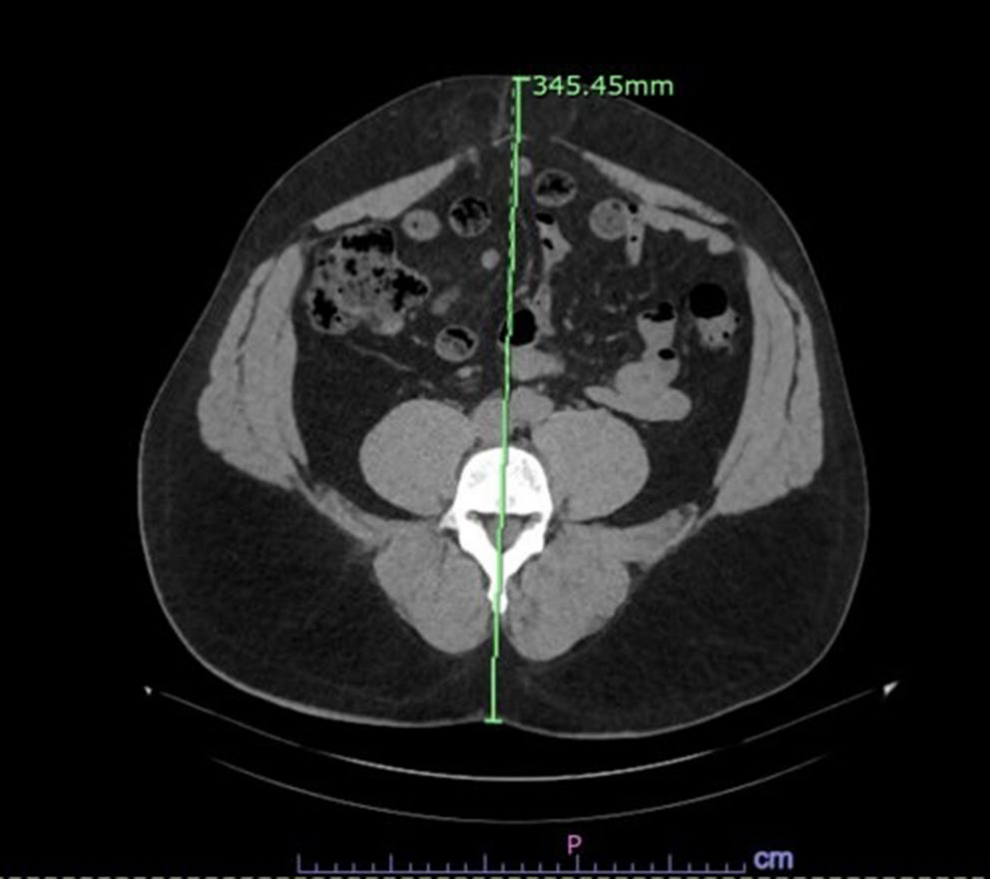
Anterior-posterior diameter (APD) was measured at the L4 vertebral level using the distance from the anterior abdominal skin to the back skin while maintaining parallelalignment with the L4 spinous process on axial slices.

Anterior-posterior diameter (APD) was measured in the PACS (Picture Archiving and Communication System), on preoperative abdomen/pelvis CT scans by two blinded board-eligible radiologists to evaluate for concordance. The distance in centimeters from the skin of the midline back to the skin of the anterior abdomen was measured using the ruler tool at the level of the L4 vertebral body, a landmark that has been validated as the standard site for quantifying abdominal adiposity on CT. Prior work established that a single axial slice at this level reliably reflects overall body fat distribution (Tokunaga et al.; Matsuzawa et al.; Sjöström et al.; Kvist et al.), while Fujioka et al. demonstrated its correlation with impaired glucose and lipid metabolism, underscoring its clinical significance [15–19]. Yoshizumi et al. further standardized the technique at L4–L5 for reproducibility, and more recent studies confirm its utility in predicting cardiometabolic risk [21, 22]. Taken together, these data support the use of L4 as a reproducible and clinically meaningful reference point for assessing abdominal wall thickness. The plane of the measurement was oriented parallel to the spinous process, with the ruler passing through the midline of the vertebral body (Fig. 1). After an initial calibration to assess inter-reader agreement, two radiologists independently verified the measurements.

### Statistical analysis

To establish the APD threshold associated with a significant increase in hernia recurrence, a Classification and Regression Tree (CART) analysis was initially performed [11]. Then patients were divided to two groups based on their APD below or above the identified threshold. Bivariate analyses further explored the difference between the preoperative characteristics of the two groups and the relationship between those factors and recurrence. Further bivariate sub-group analyses were performed to identify how the APD threshold affected the recurrence rate by both hernia type (incisional vs. primary ventral) as well as by sex (male vs. female). Lastly, all preoperative risk factors that were associated with recurrence with *p* < 0.1 in the initial bivariate were entered into a Cox regression model to determine independent risk factors for recurrence as a time-to-recurrence model. Based on the threshold for APD found in the CART analysis, a Receiver Operating Characteristic (ROC) curve analysis was then conducted using Z-test to compare the predictive ability for recurrence risk (Area Under the Curve, AUC) of APD and the BMI of 33.67, as established by our previous study. A Kaplan-Meier survival analysis assessed recurrence-free survival over a five-year follow-up period. Continuous variables were compared using Student’s t-test, while categorical variables were analyzed using Pearson’s chi-squared and Fisher’s exact tests, as appropriate. Statistical significance was determined using a p-value threshold of ≤ 0.05. SPSS, version 28.0 (IBM Corp, Armonk, NY) **was utilized.**

## Results

Two hundred and sixty-seven patients underwent elective hernia repair during the study period. The mean age of the cohort was 53.39 ± 12.1 years, and 62.5% (*N* = 167) were male. Among these patients 207 (77.5%) were Hispanic, 23 (8.6%) were Black, 13 (4.8%) were Non-Hispanic White, 3 (1.1%) were Asian, and 12 (4.4%) did not specify race.

The mean BMI was 32.42 ± 4.94 (kg/m²) and the mean APD at L4 was 27.67 ± 4.14 cm. Open hernia repair accounted for 81.3% (*N* = 210) of the repairs done. Mesh was utilized in 95.3% of cases (*N* = 246) with 85.0% of cases (*N* = 228) using permanent mesh and 6.3% of cases (*N* = 17) using biologic mesh. The overall recurrence rate was 10.5% (*N* = 28) over a median follow- up interval of 946 days. The median time from ventral hernia repair to recurrence was 734 days. CART analysis identified an APD of 29.7 cm as the threshold for recurrence. There was no statistically significant difference in burden of comorbidities (Table 1) or defect size, percentage of recurrent hernias, mesh use, mesh type, or operative method between the APD ≤ 29.7 cohort and APD > 29.7 cohort. (Table 2)

**Table 1:** Comparison of anterior-to-posterior depth (APD) threshold value for hernia recurrence in regards to patient demographics and pre-existing conditions

Table 1: Demographics within APD Groups			
	APD < 29.7 cm (*n* = 188, 72.8%) (n%)	APD > 29.7 cm (*n* = 70, 27.2%) (n%)	*p* value
Age (years)			0.914
Mean ± SD	53.39 ± 12.1	53.57 ± 10.4	
**BMI (kg/m2)**			< 0.001
Mean ± SD	30.68 ± 3.1	36.9 ± 5.9	
**APD (cm)**			< 0.001
Mean ± SD	25.8 ± 2.7	32.6 ± 3.1	
**Gender**			0.518
Male	67 (35.6)	28 (40)	
Female	121 (64.4)	42 (60)	
**Race/Ethnicity**			0.099
Non-hispanic white	7 (3.7)	6 (8.6)	
Black	13 (6.9)	10 (14.3)	
Hispanic	155 (82.4)	52 (74.3)	
Asian	3 (1.6)	0 (0.0)	
Other	10 (5.3)	2 (2.9)	
**Smoking**	26 (13.8)	9 (12.9)	0.839
**Diabetes**	44 (23.4)	23 (32.9)	0.124
**Hypertension**	83 (44.1)	40 (57.1)	0.063
**CV Disease**	15 (8.0)	7 (10.0)	0.605
**Pulm Disease**	14 (7.4)	6 (8.6)	0.764
**CKD**	12 (6.4)	3 (4.3)	0.537
**Cirrhosis**	4 (2.1)	2 (2.9)	0.731

**Table 2:** Comparison of anterior-to-posterior depth (APD) threshold value for hernia recurrence in regards to hernia characteristics and operative factors

Table 2: Hernia Characteristics in within APD Groups			
	APD < 29.7 cm (*n* = 188, 72.8%) (n%)	APD > 29.7 cm (70, 27.2%) (*n*%)	*p* value
Fascial Defect (cm)			0.951
Mean ± SD	7.3 ± 4.7	7.4 ± 4.3	
**Incarcerated**			< 0.001
No	144 (76.6)	39 (55.7)	
Yes	44 (23.4)	31 (44.3)	
**Recurrent**			0.059
No	143 (76.1)	45 (64.3)	
Yes	45 (23.9)	25 (35.7)	
**Operative Method**			0.071
Open Repair	148 (78.7)	62 (88.6)	0.071
Laparoscopic Repair	40 (21.3)	8 (11.4)	0.071
**Mesh Used**			0.901
No	9 (4.8)	3 (4.3)	
Yes	179 (95.2)	67 (95.7)	
**Mesh Type**			0.339
None	9 (4.8)	3 (4.3)	
Permanent	164 (87.2)	64 (92.8)	
Biologic	15 (8.0)	2 (2.9)	
**Mesh Location**			0.222
Onlay	53 (29.4)	16 (24.2)	
Retrorectus	32 (17.8)	10 (15.2)	
Underlay	59 (32.8)	31 (47.0)	
Intraperitoneal	36 (20.0)	9 (13.6)	
**Drain**			0.181
No	103 (56.3)	36 (51.4)	
Yes	80 (43.7)	34 (48.6)	
**Bowel Resection**			0.391
No	186 (98.9)	69 (100.0)	
Yes	2 (1.1)	0 (0.0)	

On bivariate analysis, APD of > 29.7 was found to be significantly associated with hernia recurrence (18.6% vs. 7.4%, *p* < 0.001). Additionally, there was a statistically significant difference in hernia recurrence with BMI (34.4 ± 8.2 kg/m^2^ vs. 32.1 ± 4.3 kg/m^2^, *p* < 0.001). The only other significant factor in the bivariate analysis for risk of hernia recurrence was a prior failed hernia repair with a recurrent hernia (17% vs. 8.1%, *p* = 0.034). Defect size, incarcerated bowel, open surgery, type of mesh used, mesh location, presence of a drain, and a history of COPD, CKD, cirrhosis, diabetes, and smoking were not associated with increased risk of hernia. (Table 3)

**Table 3:** Comparison of patients with hernia recurrence in regards to patient demographics and pre-existing conditions

Table 3: Demographics of Hernia Recurrence			
	Recurred (*N* = 28)%	No Recurrence (*N* = 239)	*p* value
Age (years)			0.914
Mean ± SD	55.18 ± 10.29	52.95 ± 11.86	
**BMI (kg/m2)**			0.010
Mean ± SD	34.48 ± 8.29	32.18 ± 4.35	
**APD (cm)**			0.030
Mean ± SD	29.09 ± 5.44	27.50 ± 3.95	
**Gender**			0.840
Male	18 (64.3)	149 (62.3)	
Female	10 (35.7)	90 (37.7)	
**Race/Ethnicity**			0.483
Non-hispanic white	2 (7.1)	12 (5.0)	
Black	1 (3.6)	23 (9.6)	
Hispanic	25 (89.3)	188 (78.7)	
Asian	0 (0.0)	3 (1.3)	
Other	0 (0.0)	13 (5.4)	
**Smoking**	3 (10.7)	35 (14.6)	0.573
**Diabetes**	5 (17.9)	63 (26.4)	0.329
**Hypertension**	14 (50.0)	112 (46.9)	0.753
**CV Disease**	4 (14.3)	19 (7.9)	0.258
**Pulm Disease**	2 (7.1)	18 (7.5)	0.941
**CKD**	2 (7.1)	13 (5.5)	0.715
**Cirrhosis**	2 (7.1)	4 (1.7)	0.065

Among female patients, those with an APD > 29.7 cm experienced a significantly higher rate of hernia recurrence (21.4% vs. 4.5%, *p* = 0.018). In male patients, recurrence was also more frequent in the APD > 29.7 cm group (16.7% vs. 9.1%, *p* = 0.25). (Table 4)

**Table 4:** Comparison of patients with hernia recurrence in regards hernia characteristics and operative factors

Table 4: Hernia Characteristics of patients with Recurrence			
	Recurred (*N* = 28)%	No Recurrence (*N* = 239)	*p* value
Fascial Defect (cm)			0.372
Mean ± SD	7.42 ± 6.19	7.28 ± 4.40	
**Incarcerated**			0.683
No	19 (67.9)	171 (71.5)	
Yes	9 (32.1)	68 (28.5)	
**Recurrent**			0.034
No	16 (57.1)	181 (75.7)	
Yes	12 (42.9)	58 (24.3)	
**Operative Method**			0.493
Open Repair	24 (85.7)	192 (80.3)	
Laparoscopic Repair	4 (14.3)	47 (19.7)	
**Mesh Used**			0.170
No	3 (10.7)	11 (4.6)	
Yes	25 (89.3)	228 (95.4)	
**Mesh Type**			0.197
None	3 (10.7)	9 (3.8)	
Permanent	24 (85.7)	211 (88.7)	
Biologic	1 (3.6)	18 (7.6)	
**Mesh Location**			0.783
Onlay	7 (28.0)	62 (27.0)	
Retrorectus	6 (24.0)	40 (17.4)	
Underlay	7 (28.0)	85 (37.0)	
Intraperitoneal	5 (20.0)	43 (18.7)	
**Drain**			0.181
No	103 (56.3)	36 (51.4)	
Yes	80 (43.7)	34 (48.6)	
**Bowel Resection**			0.068
No	27 (96.4)	237 (99.6)	
Yes	1 (3.6)	1 (0.4)	

When stratified by hernia type, recurrence rates were similar between incisional hernias (10.0% vs. 10.7%, *p* = 1.00). Among patients with incisional hernias, those with an APD > 29.7 cm had a higher recurrence rate (20.6% vs. 9.2% *p* = 0.15), although this difference did not reach statistical significance. In contrast, among patients with primary ventral hernias, an APD > 29.7 cm was significantly associated with recurrence (26.1%vs. 2.5%, *p* = 0.037).

Both APD and recurrent hernia were included in the Cox regression analysis. APD > 29.7 cm (hazard ratio (HR) of 2.33, 95% CI 1.09:4.98, *p* = 0.029) was independently associated with increased risk of hernia recurrence. A recurrent hernia was not independently associated with recurrence (HR 1.34, 95% CI 0.62: 2.90, *p* = 0.44).

**Fig. 2:**
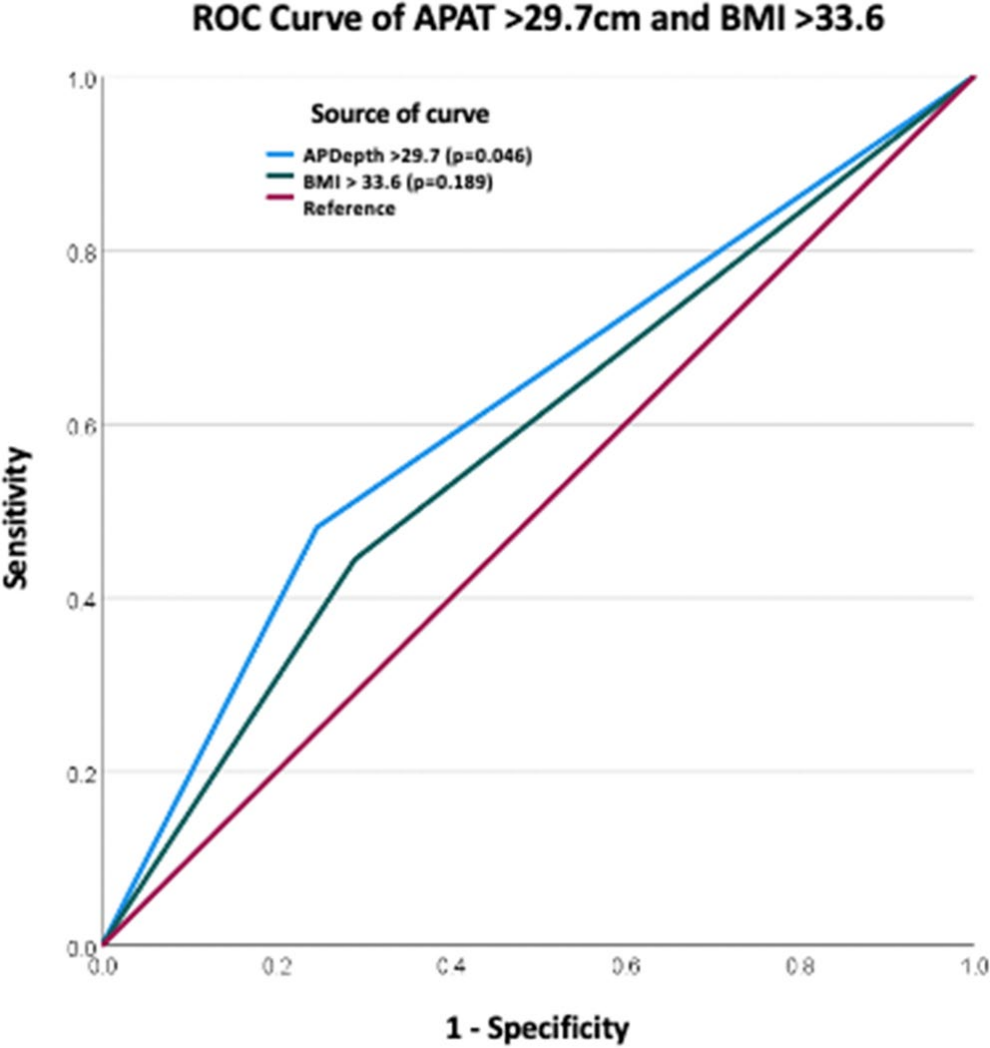
ROC curve of APD > 29.7 cm and BMI showing superior AUC for APD of 0.6117compared to BMI > 33.67, 0.57, p = 0.18

**Fig. 3:**
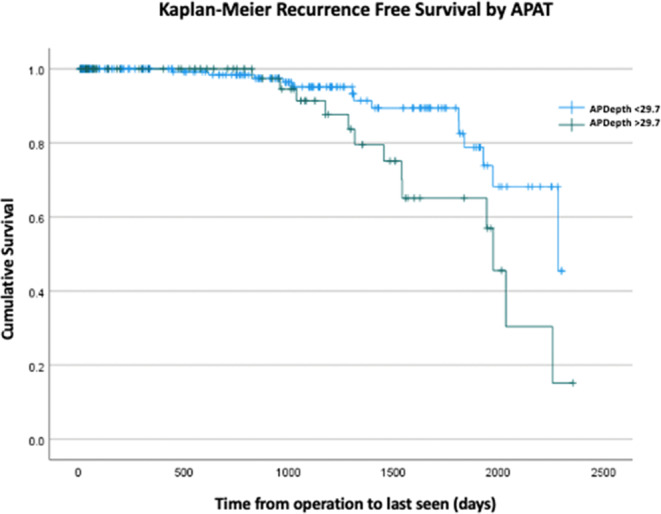
Kaplan–Meier curve showing a stepwise decrease in hernia recurrence for APD < 29.7 compared to APD > 29.7 Precis: This study identifies anterior-posterior depth (APD) > 29.7 cm as a predictor of ventral hernia recurrence, outperforming BMI in predictive accuracy. APD independently correlates with recurrence risk, emphasizing the need for refined preoperative risk assessment. Integrating APD measurement into practice could enhance patient selection and improve surgical outcomes in hernia repair

Kaplan-Meier analysis demonstrated that patients with an APD ≤ 29.7 cm had significantly higher five-year recurrence-free rates compared to those with APD > 29.7 cm (Recurrence Free Rate (RFR) 0.70, SE 0.09 vs. 0.37 SE 0.13, *p* = 0.02); corresponding Recurrence 0.30 vs. 0.63, 95% CI 0.12:0.48 vs. 0.38:0.88; *p* < 0.02) (Fig. 2). Mean time to recurrence was substantially increased in the APD ≤ 29.70 (2081.41 days, 95% CI 1978.34:2184.48 vs. 1841.70 days, 95% CI 2026.96:1974.00, *p* = 0.02). Finally, a ROC Curve Analysis showed that the AUC for APD > 29.7 cm and BMI > 33.67 were 0.617 [SE 0.06 (95%CI 0.499:0.735, *p* = 0.046)] and 0.577 [SE 0.06 (95%CI 0.46:0.69, *p* = 0.18)] respectively (Fig. 3).

## Discussion

In this study, we identified an APD threshold of > 29.7 cm as a key predictor of hernia recurrence in patients undergoing elective ventral hernia repair with defects of 4 cm or greater. This finding underscores the significance of central adiposity in surgical outcomes. APD demonstrated superior predictive accuracy (AUC = 0.61, *p* = 0.04) compared to BMI (AUC = 0.57, *p* = 0.18) when using a BMI threshold of 33.67. While the AUC of 0.61 reflects modest discriminatory ability, this is expected when evaluating a single anatomic variable rather than a multivariable prediction model. Because APD is used as a risk factor rather than a standalone diagnostic classifier, the clinical utility lies in its independent association with recurrence and its superiority over BMI in predicting long-term outcomes. Patients with APD > 29.7 cm had a 37% 5-year recurrence-free survival, significantly lower than the 70% observed in patients with APD ≤ 29.7 cm highlighting the importance of preoperative assessment beyond traditional BMI metrics.

Our finding that APD is independently associated with ventral hernia recurrence is consistent with a growing body of evidence linking BMI, visceral adiposity, and increased morbidity after hernia repair including recurrence [12–14, 23–26]. Previous literature looking at BMI, has found it to be implicated in several adverse post-operative outcomes. Liang et al.’s systematic review [23] established that obesity contributed to increased post-operative complications, reinforcing the necessity for individualized surgical risk assessment for patients with a BMI 30–50 kg/m2. In an analysis of an institutional cohort undergoing hernia repair published from our group, Cook et al. [11] found a threshold of BMI > 33.67 was independently associated with hernia recurrence. Similarly in an analysis of 90,000 patients in the NSQIP database, Mabeza et al. [27] found a BMI > 32 as the threshold for perioperative morbidity. In our subgroup analyses, we observed that the impact of APD on recurrence was directionally consistent across hernia types and sexes. Among patients with primary ventral hernias, an APD > 29.7 cm was strongly associated with recurrence, while a similar trend was observed in patients with incisional hernias, although not statistically significant, likely reflecting limited power.

In prior literature, incisional hernias often demonstrate higher recurrence rates than primary ventral hernias, largely due to factors such as scarred tissue planes, impaired vascularity, and altered biomechanics at prior incision sites. In our cohort, however, recurrence rates between ventral and incisional hernias were similar, and we believe this is primarily related to our inclusion criteria. By restricting the study to defects ≥ 4 cm (in either dimension), we selected for a population with larger, higher-risk hernias regardless of etiology. Larger primary ventral hernias begin to behave more similarly to incisional hernias with respect to tissue quality, tension characteristics, and the technical complexity of repair. In other words, the size threshold likely attenuated the typical difference in recurrence risk seen between the two groups by creating a more homogeneous cohort of clinically significant hernias. Additionally, because repairs were performed in a safety-net population with variable follow-up and substantial comorbid burden, systemic and patient-related factors may have contributed more strongly to recurrence risk than the distinction between primary versus incisional origin. Thus, the minimal defect size requirement likely narrowed the expected difference in recurrence rates by selecting for larger, more complex hernias in both categories.

Likewise, APD > 29.7 cm was associated with a markedly increased risk of recurrence in women, and a similar but nonsignificant association was seen in men. These findings suggest that APD may serve as a generalizable marker of recurrence risk, with its predictive value potentially differing across patient subgroups. Taken together with prior evidence linking BMI to postoperative morbidity and recurrence, our results highlight the role of central adiposity in shaping outcomes after ventral hernia repair.

Prior studies have used various imaging-based methods to quantify visceral adiposity and assess its impact on surgical outcomes. For example, Winters et al. [13] analyzed preoperative CT scans, measuring visceral fat area (VFA) and subcutaneous fat area (SFA) to assess their correlation with hernia recurrence after component separation. Similarly, Aquina et al. [14] used visceral fat quantification on CT to demonstrate that visceral obesity, rather than BMI alone, was strongly associated with incisional hernia formation following colorectal surgery. These findings highlight the limitations of BMI as a surrogate for obesity-related risk stratification in hernia surgery. Our results build on these findings by suggesting that central adiposity, as measured by APD, plays a more critical role in hernia recurrence than overall BMI.

This study extends existing knowledge within the current literature by identifying an imaging-based threshold that predicts long-term hernia repair outcomes. Aquina et al. [14] in a retrospective analysis of colectomies with primary closure of the abdominal wall found that visceral obesity measured on preoperative CT scan had a HR 2.04 risk for incisional hernia. Both Tanaka [28] and Sabbagh [29] utilized CT scans to volumetrically analyze the hernia sac of incisional hernias to accurately predict recurrence, but only in patients with large hernias > 10 cm with loss of domain. However, Kumar et al. in 2022 [30], in a validation study found that CT scoring via Tanaka and Sabbagh methods did not correlate with recurrence; although, this study only measured hernia recurrence at 1 year. Several other measurements have previously been analyzed in the literature and found to be non-correlative. Blair et al. [31] found that abdominal wall thickness at the umbilicus and defect size had no association with recurrence; however, this study was underpowered with only 4 recurrent hernias. In a large systematic review and meta-analysis, Parker et al. [32] showed that hernia width and area measured via CT scan were not predictive of a recurrent hernia. Our study uniquely contributes to this literature by establishing APD as a more precise and practical metric than BMI for assessing abdominal obesity and predicting long-term recurrence, particularly in defects > 4 cm. Additionally, we demonstrate that hernia recurrence can persist up to five years postoperatively, emphasizing the need for extended follow-up in high-risk patients.

APD may play a critical role in the biomechanics of the abdominal wall and its ability to withstand intra-abdominal pressure. According to the Law of Laplace, wall tension is directly proportional to the pressure within a cylinder and the radius of that structure. In the context of the abdomen, as visceral adiposity increases and APD expands, the abdominal wall is subjected to greater tension, which can predispose to hernia formation and recurrence. Given that BMI alone fails to differentiate between visceral adiposity and other fat distributions, APD may provide a more precise metric for assessing abdominal wall stress and recurrence risk. Understanding the interplay between APD, intra-abdominal pressure, and hernia recurrence is crucial for optimizing preoperative risk stratification and improving long-term surgical outcomes.

Given the significance of these findings, APD could be easily integrated into preoperative evaluation protocols for patients who undergo elective ventral hernia repair and could be measured by surgeons at the initial clinic visit at the preoperative visit. Preoperative weight loss should be encouraged if patients are above the APD threshold of 29.7 cm to mitigate recurrence risk given that an APD of 29.7 cm was independently associated with hernia recurrence. Moreover, given the increased abdominal wall tension associated with APD > 29.7 cm, additional steps beyond weight loss could be considered to decrease recurrence risk, such as preoperative botulinum toxin injection or intraoperative fascial release to increase abdominal wall compliance. Post-operative surveillance for patients with APD > 29.7 cm should also have a higher suspicion of recurrence and weight loss should be encouraged (potentially with a GLP-1) given the longitudinal risk for hernia recurrence over 5 years in this study. Prior research, such as Bhardwaj et al. [33], demonstrated a 5-year recurrence rate that was greater than 40% with mesh and 70% in patients without mesh. Our Kaplan-Meier analysis showed a decreased recurrence rate of 37% at 5 years for BMI ≤ 29.7 cm, reinforcing the critical need for optimizing patients preoperatively to improve outcomes. Implementing APD measurements into routine preoperative screening for patients with available CT scans would be a simple, objective way to enhance surgical decision-making and patient counseling.

Despite its strengths, this study has several limitations including its retrospective design causing a potential for selection and information bias. This study only contained 267 patients with only 28 recurrences, which may affect the statistical significance of the study population, especially in the subgroup analyses. This increases the potential for Type II error for these results. Additionally, recurrence rates may be underestimated due to reliance on patient follow-up in emergency or outpatient settings, and this may also explain our longer median hernia time to recurrence about two years (734 days). Although follow-up was not fully standardized, most patients were seen at approximately 2 weeks, 6 months, 1 year, and then annually. Therefore, the timing of recurrence reflects when it was detected within the hospital system, not necessarily when the patient first experienced symptoms. This was also a cohort study from a large, diverse county healthcare system, offering insights into an underserved and underrepresented patient population. The cohort included a high proportion of Latino patients, reflecting the demographic composition of one of the largest county healthcare systems in the United States and the fastest growing ethnic group in the country; however, these patients may not be representative of other populations in different healthcare settings. Another limitation is the use of BMI 33.67 as the threshold for worsened recurrence outcomes which was calculated from a previous study from our group. Additionally, this study has longitudinal follow-up for up to five years post-surgery. This study specifically focuses on surgical risk after elective hernia repair, and additional factors such as the higher incidence of incarcerated hernias in the APD > 29.7 cm group (44.3% vs. 23.4%, *p* < 0.001) may have influenced outcomes, although, notably, incarcerated hernia itself was not found to be an independent risk factor for recurrence. Finally, most hernia repairs in this study were performed via an open approach, with no representation of robotic repairs, which differs from contemporary practice patterns. To confirm the value of this novel study, future prospective randomized studies are needed to validate these findings.

##  Conclusion

In our study, the use of APD CT measurements provided an objective, reproducible, and rapid method to augment preoperative evaluation for visceral obesity and the risk for hernia recurrence that was not reliant on traditional BMI, and, in fact, improved upon a simple BMI threshold. These insights emphasize the need for further research into the role of intra-abdominal fat distribution in hernia pathophysiology. Prospective validation of the APD threshold in multi-institutional studies should be performed to further validate this measurement. Additionally, further investigation in the role of weight loss, visceral fat reduction, and decreased APD should be further investigated to continue to select the most optimized patients for this surgery and to reduce their recurrence risk.

## Data Availability

The data that support the findings of this study are available from the corresponding author upon reasonable request.

## Abbreviations

APD: Anterior to posterior depth
